# Re-Evaluating Positive Airway Pressure Indications in Obstructive Sleep Apnea: A Cross-Classification Analysis of Polysomnographic Severity and the Baveno Phenotypic Classification

**DOI:** 10.3390/jcm15155767

**Published:** 2026-07-23

**Authors:** Orhan Dalkılıç, Nesrin Sarıman

**Affiliations:** 1 Department of Pulmonology, Faculty of Medicine, Uskudar University, Istanbul 34662, Turkey; 2 Department of Pulmonology, Hisar Intercontinental Hospital, Istanbul 34768, Turkey; nesrin.sariman@hisarhospital.com

**Keywords:** obstructive sleep apnea, Baveno classification, polysomnography, positive airway pressure, reclassification

## Abstract

**Background/Objectives:** Obstructive sleep apnea (OSA) is traditionally diagnosed via polysomnography (PSG) using the apnea–hypopnea index (AHI). However, AHI alone may inadequately reflect the clinical heterogeneity, symptom burden, and cardiometabolic consequences of the disease. The Baveno classification incorporates symptom severity and comorbidity burden into a multidimensional framework. We aimed to evaluate the agreement between PSG-staging and the Baveno classification, to investigate the impact of reclassification on positive airway pressure (PAP) treatment recommendations. **Methods:** This retrospective study included 395 adults with OSA diagnosed via PSG between January 2016 and June 2024. Patients were categorized according to PSG severity (mild, moderate, and severe) and Baveno phenotypes (A, B, C, D). PAP therapy indication was defined as AHI ≥ 15 events/hour in the PSG-based classification and as Baveno groups B-D in the multidimensional approach. Agreement between the systems was assessed using Cohen’s kappa coefficient. **Results:** PAP therapy was indicated in 252 patients (63.8%) via PSG and in 268 patients (67.8%) via the Baveno classification. Overall agreement between the systems was 63.5%, (κ = 0.191) and increased with disease severity, reaching 87.1% with severe OSA. Overall, 144 patients (36.5%) were reclassified, including 80 patients newly identified as candidates for PAP therapy and 64 patients reclassified as not requiring PAP therapy according to the Baveno framework. BMI showed only a weak correlation with AHI overall and no significant association in women. **Conclusions:** Combined use of PSG and the Baveno approach provides more individualized PAP treatment decisions, particularly among clinically heterogeneous patients and those within intermediate severity categories.

## 1. Introduction

Obstructive sleep apnea (OSA) is a growing global public health concern characterized by intermittent hypoxemia, sleep fragmentation, and autonomic dysfunction. In recent years, the prevalence of OSA has increased substantially due to rising obesity rates and population aging, with current estimates suggesting that nearly one billion adults worldwide may be affected by varying degrees of the disease [1,2]. In addition to impaired sleep quality and excessive daytime sleepiness, OSA is strongly associated with hypertension, atrial fibrillation, coronary artery disease, stroke, insulin resistance, and increased cardiovascular mortality [3].

Currently, the apnea–hypopnea index (AHI), measured via polysomnography (PSG), remains the gold standard for determining disease severity and guiding treatment decisions. Traditionally, OSA severity is classified as mild, moderate, or severe according to the AHI. However, the AHI primarily quantifies the mechanical frequency of respiratory events and does not fully capture the hypoxic burden, sleep disruption, or, perhaps most importantly, the cardiometabolic risk profile associated with the disease [4].

This one-dimensional approach creates important paradoxes in clinical practice. Patients with similar AHI values may exhibit substantial differences in symptom severity, cardiovascular risk, and response to positive airway pressure (PAP) therapy [5,6,7]. Consequently, patients with relatively low AHI values but marked oxygen desaturation and significant symptom burden may be at risk of undertreatment, whereas asymptomatic individuals with high AHI values may be exposed to overtreatment. Moreover, physiological parameters such as hypoxic burden and sleep fragmentation have been reported to be more strongly associated with cardiovascular outcomes than the frequency of respiratory events alone [8].

To better address this clinical heterogeneity, the Baveno classification, developed by the European Sleep Research Society, has been proposed as a multidimensional framework that stages OSA not only according to AHI but also according to symptom burden and cardiometabolic comorbidity profiles, categorizing patients into four groups (A–D) [9,10]. By jointly considering excessive daytime sleepiness and major comorbid conditions, this model aims to provide a more comprehensive characterization of disease heterogeneity and to support individualized treatment strategies. Although the prognostic value of the Baveno classification for cardiovascular outcomes has been demonstrated in previous studies, evidence regarding its agreement with PSG findings and its direct impact on PAP treatment decisions in routine clinical practice remains limited [10,11].

The primary objective of this retrospective study was to evaluate the cross-classification agreement between traditional AHI-based severity staging and the Baveno classification in a cohort of patients diagnosed with OSA via PSG. Furthermore, we aimed to assess the extent to which these two approaches can be integrated for PAP treatment decision-making and to investigate how multidimensional clinical assessment may reshape therapeutic decisions, particularly among patients within the “gray zone” of OSA severity, defined by an AHI between 5 and 15 events per hour.

## 2. Materials and Methods

### 2.1. Study Design and Population

**Figure 1:**
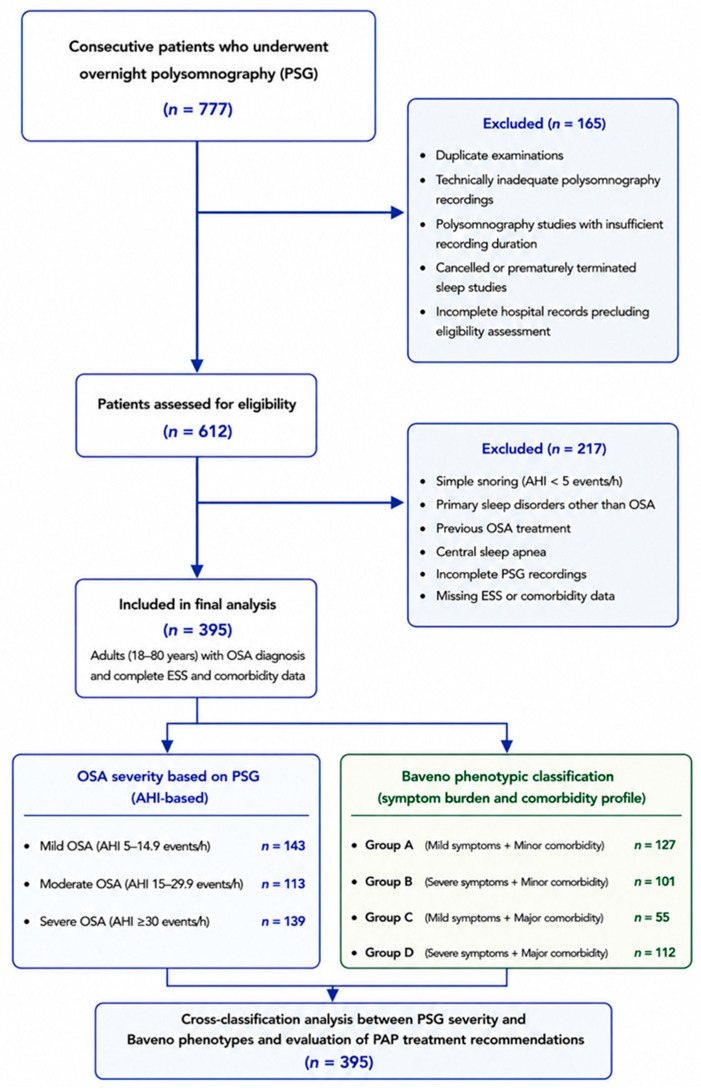
Flow diagram of patient selection and study design. Flow diagram illustrating patient selection, eligibility assessment, and study design. The final study cohort (*n* = 395) was classified according to PSG-defined OSA severity and the Baveno phenotypic classification for subsequent cross-classification and PAP treatment recommendation analyses. Abbreviations: PSG, polysomnography; OSA, obstructive sleep apnea; AHI, apnea–hypopnea index; ESS, Epworth Sleepiness Scale; PAP, positive airway pressure.

This single-center retrospective cross-sectional study was conducted at the Sleep Laboratory of Hisar Intercontinental Hospital. An overnight polysomnographic evaluation was performed using a Natus Embla SDx system, and data were acquired and analyzed using RemLogic software, version 4 (Natus Medical Inc., Middleton, WI, USA). All consecutive overnight sleep studies performed between January 2016 and June 2024 were retrospectively screened through the hospital database. During this period, 777 overnight sleep studies were identified. Duplicate examinations, technically inadequate recordings, studies with insufficient recording duration, cancelled or prematurely terminated studies, and records with insufficient clinical information for eligibility assessment were excluded, leaving 612 patients who underwent diagnostic overnight PSG for eligibility assessment (Figure 1).

Among these 612 eligible patients, individuals with simple snoring (AHI < 5 events/h), primary sleep disorders other than obstructive sleep apnea (OSA), previous OSA treatment, central sleep apnea, incomplete PSG recordings, or missing Epworth Sleepiness Scale (ESS) scores or comorbidity data were excluded. Following application of the predefined inclusion and exclusion criteria, 395 adults aged 18–80 years with a diagnosis of OSA and complete clinical information were included in the final analysis.

Demographic, clinical, and polysomnographic variables were extracted retrospectively from the electronic hospital database. The final study cohort was subsequently classified according to both conventional AHI-based PSG severity (mild, moderate, and severe OSA) and the Baveno phenotypic classification (Groups A–D) based on symptom burden and cardiometabolic comorbidity profile. Cross-classification analyses were then performed to evaluate the agreement between the two classification systems and their implications for PAP treatment recommendations.

### 2.2. Polysomnography and Baveno Classification

Based on standard PSG recordings, OSA was defined as an apnea–hypopnea index (AHI) of ≥5 events/hour. Disease severity was categorized as mild (5–14.9 events/hour), moderate (15–29.9 events/hour), or severe (≥30 events/hour). The Baveno classification was applied according to the recommendations of the European Respiratory Society (ERS) and was structured based on symptom severity and comorbidity burden. Severe symptom burden was defined as an ESS score ≥ 11. Respiratory events were scored according to the American Academy of Sleep Medicine (AASM) Scoring Manual that was current at the time of each polysomnographic study; namely, The AASM Manual for the Scoring of Sleep and Associated Events, Summary of Updates in Version 2.1 (2014) and Summary of Updates in Version 2.4 (2017). Apnea was defined as a ≥90% reduction in airflow from baseline lasting for at least 10 s. hypopnea was defined as a ≥30% reduction in airflow lasting for at least 10 s and associated with either a ≥3% oxygen desaturation from the pre-event baseline or arousal. The Apnea–Hypopnea Index (AHI) was calculated as the total number of apneas and hypopneas per hour of sleep. The oxygen desaturation index (ODI) was defined as the number of oxygen desaturation events of ≥3% per hour of sleep.

Comorbidities were defined as the presence of uncontrolled arterial hypertension, atrial fibrillation, heart failure, diabetes mellitus, or a history of stroke. If at least one of these comorbidities was present, end-organ impact was deemed major calling for an allocation to Baveno Group C or D. Uncontrolled hypertension was defined as a diagnosis of arterial hypertension accompanied by a systolic office blood pressure ≥ 140 mmHg and/or a diastolic blood pressure ≥ 90 mmHg. Minor (stable conditions and well-controlled) comorbidities are accepted for Groups A and B [11].

Accordingly, patients were classified into four phenotypic groups: Group A (ESS < 11 and minor comorbidity burden), Group B (ESS ≥ 11 and minor comorbidity burden), Group C (ESS < 11 and major comorbidity burden), and Group D (ESS ≥ 11 and major comorbidity burden).

PAP treatment indication was defined as an AHI ≥ 15 events/hour according to the conventional PSG-based approach, whereas Baveno Groups B, C, and D were considered indicative of PAP therapy under the Baveno framework, representing patients with moderate-to-high clinical risk.

### 2.3. Comparative Analysis of PSG and Baveno Classification

The primary objective of this study was to evaluate the relationship between PSG-defined OSA severity and Baveno phenotypic classification. In addition, agreement between PSG-based PAP indications and Baveno-based clinical recommendations was assessed. Cases demonstrating concordant treatment orientation between the two approaches were classified as “concordant,” whereas cases resulting in different treatment recommendations were considered to represent a “reclassification” pattern.

### 2.4. Statistical Analysis

Statistical analyses were performed using IBM SPSS Statistics version 25.0 (IBM Corp., Armonk, NY, USA). Data distribution was assessed using the Shapiro–Wilk test. Continuous variables are presented as median (interquartile range [IQRs]) according to their distribution characteristics, whereas categorical variables were expressed as frequencies and percentages.

Comparisons between groups were performed using the Median test for continuous variables and the chi-square test for categorical variables. Correlations between PSG parameters and Baveno phenotypes were evaluated using Spearman’s correlation analysis. Agreement between PSG-based PAP indications and Baveno-based recommendations was assessed using Cohen’s kappa coefficient.

To identify independent predictors of PSG–Baveno discordance, multivariable logistic regression analysis with backward elimination was performed. Statistical significance was defined as a two-sided *p*-value < 0.05.

### 2.5. Ethical Approval

The study protocol was approved by the Ethics Committee of Hisar Intercontinental Hospital (approval No. 24-8; date: 2 August 2024). Due to the retrospective study design, the requirement for written informed consent was waived by the ethics committee.

All data were anonymized and coded following the removal of personal identifiers prior to analysis. The study was conducted in accordance with the principles of the Declaration of Helsinki.

Clinical Trials: ClinicalTrials.gov ID: NCT07594288, last update posted: 14 May 2026. https://register.clinicaltrials.gov/prs/beta/studies/S000H4CV00000057/recordSummary (accessed on 15 June 2026).

### 2.6. AI Statement

ChatGPT (OpenAI, San Francisco, CA, USA) was used solely for English language editing and manuscript refinement. The authors take full responsibility for the manuscript.

## 3. Results

### Demographic and Clinical Characteristics

A total of 395 patients (322 males and 73 females) were included in the study. The median age of the cohort was 44 years, and the median apnea–hypopnea index (AHI) was 23.2 events/hour. According to conventional PSG-based severity classification, 36.2% of patients were categorized as having mild OSA, 28.6% as moderate OSA, and 35.2% as severe OSA. Based on the Baveno classification, 32.2% of the cohort were assigned to Group A, 25.6% to Group B, 13.9% to Group C, and 28.4% to Group D (Table 1 and Figure 1).

**Figure 2:**
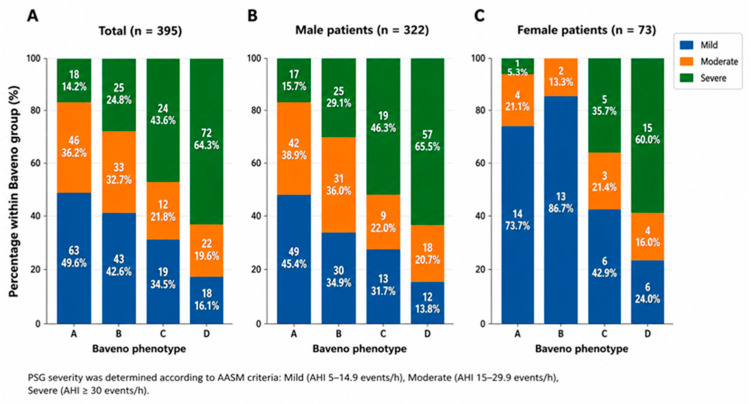
Relationship between Baveno phenotypes and PSG-defined OSA severity in the overall cohort and according to sex. Percentage distribution of PSG-defined obstructive sleep apnea (OSA) severity across Baveno phenotypes in the overall cohort (**A**), male patients (**B**), and female patients (**C**). Percentages were calculated within each Baveno phenotype. Values within the bars represent the number and percentage of patients in each PSG severity category.

Sex-stratified analyses demonstrated that the relationship between Baveno phenotype and PSG-defined OSA severity differed between men and women (Figure 2). In the overall cohort, the proportion of severe OSA increased progressively from phenotype A (14.2%) to phenotype D (64.3%), whereas the proportion of mild OSA decreased from 49.6% to 16.1%. This trend was more pronounced in male patients, in whom severe OSA increased from 15.7% in phenotype A to 65.5% in phenotype D. In contrast, female patients showed a more heterogeneous distribution, with mild OSA predominating in phenotypes A and B, while severe OSA became more frequent in phenotypes C and D.

Corresponding subgroup analyses for male and female participants are provided in the Supplementary Materials (Figures S1 and S3).

Table 2 demonstrates significant differences in polysomnographic parameters across both PSG-defined OSA severity categories and Baveno phenotypes. AHI and ODI increased progressively, whereas minimum SpO_2_ decreased with increasing disease severity (all *p* < 0.001). Similar trends were observed across Baveno groups, with the highest AHI and ODI values and the lowest minimum SpO_2_ recorded in phenotype D (all *p* < 0.001).

Using the PSG-based approach, PAP therapy was indicated in 252 patients (63.8%) based on an AHI ≥ 15 events/hour, whereas the multidimensional Baveno classification (Groups B–D) recommended PAP therapy in 268 patients (67.8%). Overall agreement between the two approaches was 63.5%, reflecting only modest concordance in clinical decision-making (Cohen’s κ = 0.191). The agreement rate increased with disease severity, rising from 44.1% among patients with mild OSA to 87.1% among those with severe OSA.

**Figure 3:**
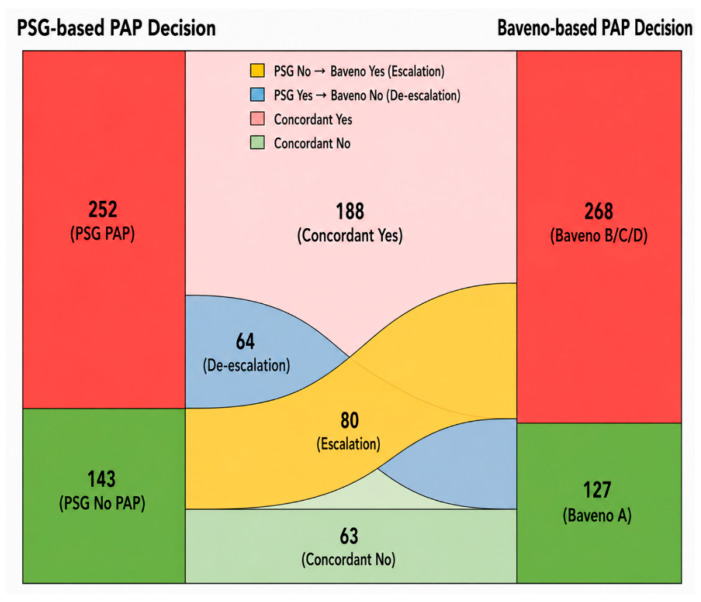
Concordance and reclassification of PAP treatment decisions between PSG and the Baveno classification. Sankey diagram showing concordant and discordant PAP treatment recommendations between PSG and the Baveno classification. Reclassification includes escalation (PSG No → Baveno Yes) and de-escalation (PSG Yes → Baveno No). Values represent the number of patients. Color coding: Yellow = Escalation (PSG No → Baveno Yes); Blue = De-escalation (PSG Yes → Baveno No); Pink = Concordant Yes; Green = Concordant No.

Overall, treatment recommendations differed in 144 patients (36.5%). The Baveno classification supported additional PAP treatment (upward reclassification) in 80 patients (20.3%) who were categorized as having mild OSA according to AHI-based PSG criteria and therefore would not have received a PAP indication under the conventional approach, but who exhibited a substantial symptom burden and/or significant comorbidities. Conversely, 64 patients (16.2%) who met the PSG criteria for PAP therapy but had a low clinical burden (Baveno Group A) were classified as not requiring PAP treatment under the Baveno framework (downward reclassification) (Figure 3 and Table 3).

Figure 3 demonstrates the agreement between PSG-based and Baveno-based PAP treatment recommendations in the total cohort and according to sex. Overall, concordant PAP recommendations were observed in 188 patients, whereas 63 patients were concordantly classified as not requiring PAP therapy. However, 80 patients were reclassified from no PAP via PSG to PAP according to the Baveno classification (escalation), while 64 patients were reclassified in the opposite direction (de-escalation). Among male patients, concordant PAP recommendations were observed in 157 patients, with 47 concordantly classified as not requiring PAP therapy. Baveno resulted in 57 escalations and 61 de-escalations, indicating a relatively balanced redistribution of treatment decisions. Among female patients, concordant PAP recommendations were observed in 29 patients, whereas 14 were concordantly classified as not requiring PAP therapy. Baveno identified 25 additional women as candidates for PAP therapy despite PSG indicating no treatment, while only 5 women were reclassified from PAP to no PAP, suggesting a greater tendency toward treatment escalation in females. Sex-specific Sankey diagrams illustrating the reclassification patterns are provided in the Supplementary Materials (Figures S2 and S4).

Bidirectional reclassification identified two clinically distinct patient subgroups (Table 3). Among the 64 patients who were down-classified from PSG-defined PAP candidates to Baveno Group A, the median AHI was 27.1 (21.8–31.0) events/h and 18 patients (28.1%) had severe OSA despite being classified as not requiring PAP because of their low symptom burden and limited cardiometabolic comorbidity. Conversely, the 80 patients who were up-classified from PSG-defined non-PAP candidates to Baveno Groups B–D all had mild OSA according to PSG (median AHI, 8.3 [6.5–11.5] events/h), but were considered PAP candidates because of clinically significant symptoms and/or cardiometabolic comorbidities.

## 4. Discussion

In the present study, we investigated the agreement between the conventional PSG-based approach, which focuses primarily on respiratory events, and the Baveno classification, a multidimensional clinical phenotyping system, in the management of obstructive sleep apnea (OSA). We also evaluated the impact of reclassification on PAP treatment decisions. The most notable finding was that treatment recommendations differed between the two approaches in approximately one-third of patients (36.5%). Although the overall agreement was 63.5%, the kappa coefficient indicated only slight agreement (κ = 0.191). This finding should not be interpreted as a methodological weakness or poor agreement; rather, it reflects the fundamentally different conceptual frameworks underlying the two classification systems. Whereas PSG primarily quantifies the physiological severity of OSA through the frequency of respiratory events, the Baveno classification focuses on clinical burden by integrating symptom severity and major cardiometabolic comorbidities [11,12]. Consequently, the observed discordance reflects clinically relevant differences between physiological severity and phenotypic disease burden, highlighting the limitations of relying on a single metric for PAP treatment decisions. These findings support a complementary rather than competitive role for the two approaches, suggesting that integrating physiological and phenotypic assessment may improve individualized PAP treatment by identifying patients who could otherwise be overlooked when either classification system is used alone.

To the best of our knowledge, this is among the few real-world studies evaluating the impact of the Baveno classification on PAP treatment decision-making through a reclassification framework.

In clinical practice, the “gray zone” of OSA severity, typically defined by an AHI between 5 and 15 events/hour, represents the subgroup in which heterogeneity is most pronounced and treatment decisions are often most challenging [13]. In the present study, the Baveno classification identified 80 patients (20.3% of the cohort) who would not have met conventional PSG criteria for PAP therapy (AHI < 15 events/hour) but who exhibited substantial symptom burden and/or significant comorbidities, thereby supporting additional PAP treatment recommendations. This upward reclassification may be clinically important because it has the potential to reduce the risk of undertreatment. Consistent with our findings, Ferraz et al. [14] reported that symptom burden may play a more influential role than comorbidities and even AHI values in determining PAP initiation in routine clinical practice, and that adherence to PAP therapy is substantially higher among symptomatic and comorbid patients (Baveno Groups C and D).

At the opposite end of the clinical spectrum, the Baveno classification suggested that PAP therapy might not be necessary in 64 patients (16.2%) who fulfilled the PSG criteria for treatment (AHI ≥ 15 events/hour) but had minimal symptom burden and limited comorbidity profiles. This subgroup had a median AHI of 27.1 (21.8–31.0) events/h, a median ODI of 24.0 (19.5–29.5) events/h, and a median minimum SpO_2_ of 81% (74.8–84.0); notably, 18 patients (28.1%) had severe OSA. Therefore, downward reclassification should not be interpreted as an automatic recommendation to withhold PAP therapy. Rather, it should prompt careful individualized clinical assessment, particularly in patients with marked nocturnal hypoxemia or severe PSG abnormalities despite limited symptoms. In addition, multinight analyses involving more than 67,000 patients by Lechat et al. [15] demonstrated that reliance on a single-night PSG may result in diagnostic and severity misclassification, particularly among patients with mild-to-moderate OSA. Accordingly, Baveno-guided downward reclassification should be viewed as a framework for integrating physiological severity with clinical burden rather than replacing PSG alone, thereby supporting more individualized and clinically balanced treatment decisions.

The agreement rate between the two classification systems increased substantially with disease severity. Concordance rose from 44.1% in mild OSA to 87.1% in severe OSA. This finding suggests that for severe OSA, physiological abnormalities—including high AHI values and profound oxygen desaturation—are more consistently reflected in clinical burden, symptom severity, and comorbidity profiles. Previous studies by Jorquera et al. [16] and Matthes et al. [17] demonstrated that Baveno Group D is associated with the highest risk of cardiovascular mortality during follow-up. The high concordance rate observed in severe OSA in our study is therefore consistent with the existing literature.

Our sex-stratified findings are consistent with growing evidence that OSA in women may be under-recognized when clinical assessment relies primarily on conventional AHI-based severity metrics. In the present cohort, women had significantly lower AHI values than men despite being older and exhibiting a higher burden of major comorbidities, suggesting that AHI alone may underestimate clinically relevant disease burden in female patients. This observation is supported by Moscucci et al. [18], who reported that women with OSA often present with atypical symptom patterns and sex-specific cardiovascular risk profiles, including a higher prevalence of heart failure with preserved ejection fraction (HFpEF) and resistant hypertension, features closely aligned with the comorbidity-driven Baveno C and D phenotypes.

These sex-related differences may also explain the lower agreement between PSG- and Baveno-based treatment recommendations observed in women. Female patients more frequently report symptoms such as fatigue, insomnia, morning headache, and mood disturbances rather than excessive daytime sleepiness, potentially reducing the sensitivity of the Epworth Sleepiness Scale for symptom assessment. Collectively, these findings suggest that multidimensional phenotypic approaches integrating symptom burden and cardiometabolic comorbidities, such as the Baveno classification, provide clinically relevant information beyond AHI alone and may be particularly valuable for improving risk stratification in women with OSA.

Nevertheless, the Baveno classification should not be regarded as a substitute for PSG. Ehab et al. [19] demonstrated that Baveno phenotypes were insufficient for predicting the CPAP titration pressures required by patients with OSA. This observation confirms that while the Baveno framework may broaden the scope of treatment indication decisions, PSG-derived physiological measurements remain essential for determining the mechanical parameters of therapy.

This study has several limitations. First, its retrospective, single-center design in a private tertiary referral sleep center may limit the generalizability of the findings because of potential referral and selection bias. Second, the relatively small number of female patients limited the statistical power of sex-stratified analyses. Third, symptom burden was assessed using the Epworth Sleepiness Scale, which may underestimate symptoms in some patients, particularly women with atypical presentations.

Finally, hypoxic burden and long-term clinical outcomes after PAP therapy were not evaluated. Prospective multicenter studies incorporating physiological markers beyond AHI,_ particularly hypoxic burden, together with multidimensional phenotypic classifications such as the Baveno framework, are needed to validate these findings, refine OSA risk stratification, and support more individualized PAP treatment decisions.

## 5. Conclusions

As increasingly emphasized in the contemporary sleep medicine literature, the future of OSA assessment is likely to move beyond AHI alone toward multidimensional scoring systems that integrate apnea burden, hypoxic burden, symptom burden, and comorbidity profiles. Within this framework, the Baveno classification should not be viewed as an alternative to conventional AHI-based assessment but rather as a complementary clinical tool capable of reducing the risks of both undertreatment and overtreatment.

The integration of the Baveno classification into routine clinical practice may improve treatment decision-making by identifying patients who are at risk of receiving either insufficient or unnecessary therapy. Such an approach represents an important step toward truly personalized management of obstructive sleep apnea.

In summary, comparison of AHI-based PSG staging with the Baveno classification revealed differences in PAP treatment indications in approximately one-third of patients (36.5%). Prospective studies evaluating treatment outcomes in patients whose management is guided by these reclassification strategies are warranted in order to determine the clinical utility and long-term effectiveness of this approach.

## Figures and Tables

**Table 1:** Baseline demographic, clinical, and polysomnographic characteristics stratified by sex.

Parameter	Total *n* = 395	Male *n* = 322	Female *n* = 73	*p* Value
Age, years	44.0 (37.0–54.0)	43.0 (36.0–52.0)	54.0 (44.0–64.0)	<0.001
BMI, kg/m^2^	30.0 (27.1–33.9)	29.7 (27.1–33.5)	31.6 (27.4–36.1)	0.057
AHI, events/hour	23.2 (9.9–38.5)	24.1 (10.7–38.7)	13.7 (7.8–34.1)	0.034
Minimum SpO_2_, %	82.0 (75.0–85.0)	81.0 (75.0–85.0)	83.0 (75.0–86.0)	0.444
OSA Severity (AHI-based), *n* (%)				
Mild (5–14.9)	143 (36.2)	104 (32.3)	39 (53.4)	-
Moderate (15–29.9)	113 (28.6)	100 (31.1)	13 (17.8)	-
Severe (≥30)	139 (35.2)	118 (36.6)	21 (28.8)	-
Severe symptoms, *n* (%)	213 (53.9)	173 (53.7)	40 (54.8)	0.972
Major comorbidity, *n* (%)	167 (42.3)	128 (39.8)	39 (53.4)	0.045
PAP indication (AHI ≥ 15), *n* (%)	252 (63.8)	218 (67.7)	34 (46.6)	0.001
BAVENO Phenotypes, *n* (%)				
A	127 (32.2)	108 (33.5)	19 (26.0)	-
B	101 (25.6)	86 (26.7)	15 (20.5)	-
C	55 (13.9)	41 (12.7)	14 (19.1)	-
D	112 (28.4)	87 (27.0)	25 (34.2)	-
PAP indication (BAVENO B, C, D), *n* (%)	268 (67.8)	214 (66.5)	54 (74.0)	0.211

BMI, body mass index; AHI, apnea–hypopnea index; SpO_2_, oxygen saturation; OSA, obstructive sleep apnea; PAP, positive airway pressure. Continuous variables are presented as medians (interquartile range), whereas categorical variables are expressed as numbers (percentages). Comparisons between groups were performed using the Median and χ^2^ tests, as appropriate.

**Table 2:** Comparative distribution of polysomnographic parameters across PSG-defined OSA severity and Baveno phenotypes.

PSG-Defined Severity Groups					
Parameter	Mild *n* = 143	Moderate *n* = 113	Severe *n* = 139	*p-*value	
AHI, events/hour	8.0 (6.4–10.7)	23.4 (20.2–26.2)	48.2 (37.8–65.8)	-	
ODI, events/hour	7.1 (5.6–9.9)	22.7 (18.4–25.0)	44.7 (35.1–61.5)	<0.001	
Minimum SpO_2_, %	85.0 (82.0–88.0)	82.0 (76.0–85.0)	75.0 (65.0–81.5)	<0.001	
Baveno phenotypes					
Parameter	A *n* = 127	B *n* = 101	C *n* = 55	D *n* = 112	*p*-value
AHI, events/hour	15.6 (8.0–27.1)	20.0 (8.2–29.7)	22.8 (10.9–44.2)	38.5 (24.1–61.4)	<0.001
ODI, events/hour	13.8 (7.0–24.8)	17.9 (6.9–30.0)	22.8 (10.7–43.4)	36.9 (23.0–57.8)	<0.001
Minimum SpO_2_, %	83.0 (78.0–86.0)	83.0 (78.0–87.0)	82.0 (75.0–85.0)	77.0 (69.0–83.0)	<0.001

AHI, apnea–hypopnea index; ODI, oxygen desaturation index; SpO_2_, oxygen saturation; PSG, polysomnography. Data are presented as medians (interquartile range). Comparisons across groups were performed using the Median test. Statistical comparisons were not performed for AHI across the PSG-defined severity groups because these categories were established based on AHI thresholds.

**Table 3:** Clinical characteristics of patients with downward and upward reclassification of PAP treatment decisions.

Variable	De-Escalation (Downward Reclassification) *n* = 64	Escalation (Upward Reclassification) *n* = 80
Age, years	39.0 (33.8–46.3)	48.0 (38.0–58.5)
BMI, kg/m^2^	28.4 (26.2–31.0)	31.6 (27.2–34.1)
AHI, events/h	27.1 (21.8–31.0)	8.3 (6.5–11.5)
ODI, events/h	24.0 (19.5–29.5)	7.4 (5.6–10.9)
Minimum SpO_2_, %	81 (74.8–84.0)	85 (82–88)
Mild OSA	—	80 (100%)
Moderate OSA	46 (71.9%)	—
Severe OSA	18 (28.1%)	—

Continuous variables are presented as medians (IQRs) and categorical variables as numbers (%). Comparison of demographic and polysomnographic characteristics between patients with downward (PSG PAP-positive → Baveno PAP-negative) and upward (PSG PAP-negative → Baveno PAP-positive) reclassification.

## Data Availability

The data presented in this study are available from the corresponding author on reasonable request, subject to institutional and ethical restrictions.

## Supplementary Materials

The following supporting information can be downloaded at: https://www.mdpi.com/article/10.3390/jcm15155767/s1, Figure S1. Supplementary Male Data for Figure 2—Baveno phenotypes and PSG-defined OSA severity; Figure S2. Supplementary Male Data for Figure 3—Sankey diagram; Figure S3. Supplementary Female Data for Figure 2—Baveno phenotypes and PSG-defined OSA severity; Figure S4. Supplementary Female Data for Figure 3—Sankey diagram.

## Abbreviations

The following abbreviations are used in this manuscript:

Abbreviation	Definition
AHI	Apnea—hypopnea index
BMI	Body mass index
ESS	Epworth Sleepiness Scale
ODI	Oxygen desaturation index
OSA	Obstructive sleep apnea
PAP	Positive airway pressure
PSG	Polysomnography
SpO2	Peripheral oxygen saturation

## References

[1] O’donnell C., O’mahony A.M., McNicholas W.T., Ryan S. (2021). Cardiovascular manifestations in obstructive sleep apnea: Current evidence and potential mechanisms. Pol. Arch. Intern. Med..

[2] Senaratna C.V., Perret J.L., Lodge C.J., Lowe A.J., Campbell B.E., Matheson M.C., Hamilton G.S., Dharmage S.C. (2017). Prevalence of obstructive sleep apnea in the general population: A systematic review. Sleep Med. Rev..

[3] Yeghiazarians Y., Jneid H., Tietjens J.R., Redline S., Brown D.L., El-Sherif N., Mehra R., Bozkurt B., Ndumele C.E., Somers V.K. (2021). Obstructive sleep apnea and cardiovascular disase: A scientific statement from the American Heart Association. Circulation.

[4] Guo J., Xiao Y. (2023). New metrics from polysomnography: Precision medicine for OSA interventions. Nat. Sci. Sleep.

[5] Izci B., Ardic S., Firat H., Sahin A., Altinors M., Karacan I. (2008). Reliability and validity studies of the Turkish version of the Epworth Sleepiness Scale. Sleep Breath.

[6] Rosales W., Vanka S.C., Singh H., Bhamrah P., Bhamrah M., Ghildiyal N., Liendo C., Asghar S., Alexander J.S., Chernyshev O.Y. (2025). Clinical Phenotypes of Obstructive Sleep Apnea: A Decade of Evidence Toward Personalized Management. Pathophysiology.

[7] Cohen O., Kundel V., Robson P., Al-Taie Z., Suárez-Fariñas M., Shah N.A. (2024). Achieving Better Understanding of Obstructive Sleep Apnea Treatment Effects on Cardiovascular Disease Outcomes through Machine Learning Approaches: A Narrative Review. J. Clin. Med..

[8] Parekh A. (2024). Hypoxic burden—Definitions, pathophysiological concepts, methods of evaluation, and clinical relevance. Curr. Opin. Pulm. Med..

[9] Randerath W., Verbraecken J., de Raaff C.A., Hedner J., Herkenrath S., Hohenhorst W., Jakob T., Marrone O., Marklund M., McNicholas W.T. (2021). European Respiratory Society guideline on non-CPAP therapies for obstructive sleep apnoea. Eur. Respir. Rev..

[10] Kapur V.K., Auckley D.H., Chowdhuri S., Kuhlmann D.C., Mehra R., Ramar K., Harrod C.G. (2017). Clinical Practice Guideline for Diagnostic Testing for Adult Obstructive Sleep Apnea. J. Clin. Sleep Med..

[11] Randerath W.J., Herkenrath S., Treml M., Grote L., Hedner J., Bonsignore M.R., Pépin J.L., Ryan S., Schiza S., Verbraecken J. (2021). Evaluation of a multicomponent grading system for obstructive sleep apnoea: The Baveno classification. ERJ Open Res..

[12] Sousa S., Caldeira J., Moita J. (2022). Beyond Apnea-Hypopnea Index: How clinical and comorbidity are important in obstructive sleep apnea. Adv. Respir. Med..

[13] Patil S.P., Ayappa I.A., Caples S.M., Kimoff R.J., Patel S.R., Harrod C.G. (2019). Treatment of Adult Obstructive Sleep Apnea with Positive Airway Pressure: An American Academy of Sleep Medicine Clinical Practice Guideline. J. Clin. Sleep Med..

[14] Ferraz M.B.D., Faria N., Correia F., Silva B., Lacerda C. (2023). Baveno classification—Rethinking obstructive sleep apnea treatment. ERJ Open Res..

[15] Lechat B., Naik G., Reynolds A., Aishah A., Scott H., Loffler K.A., Vakulin A., Escourrou P., McEvoy R.D., Adams R.J. (2022). Multinight Prevalence, Variability, and Diagnostic Misclassification of Obstructive Sleep Apnea. Am. J. Respir. Crit. Care Med..

[16] Jorquera J., Dreyse J., Salas C., Letelier F., Weissglas B., Del-Río J., Henríquez-Beltrán M., Labarca G., Jorquera-Díaz J. (2023). Clinical Application of the Multicomponent Grading System for Sleep Apnea Classification and Incident Cardiovascular Mortality. Sleep Sci..

[17] Matthes S., Treml M., Grote L., Hedner J., Zou D., Bonsignore M.R., Pépin J.-L., Bailly S., Ryan S., McNicholas W.T. (2024). The modified Baveno classification for obstructive sleep apnoea: Development and evaluation based on the ESADA database. Eur. Respir. J..

[18] Moscucci F., Bucciarelli V., Gallina S., Sciomer S., Mattioli A.V., Maffei S., Nodari S., Pedrinelli R., Andreozzi P., Basili S. (2025). Gender Cardiovascular Disease Study Group of the Italian Society of Cardiology (SIC); Gender Working Group of the Italian Society of Internal Medicine (SIMI). Obstructive sleep apnea syndrome (OSAS) in women: A forgotten cardiovascular risk factor. Maturitas.

[19] Ehab A., Kempa A.T., Englert H., Bittar S.A., Yousef A.M., Abdelwahab H.W. (2023). The Baveno Classification as a Predictor of CPAP Titration Pressure in Obstructive Sleep Apnea Syndrome. Adv. Respir. Med..

